# Study of the Fabrication Technology of Hybrid Microfluidic Biochips for Label-Free Detection of Proteins

**DOI:** 10.3390/mi13010020

**Published:** 2021-12-24

**Authors:** Nikita Sitkov, Tatiana Zimina, Alexey Kolobov, Evgeny Sevostyanov, Valentina Trushlyakova, Viktor Luchinin, Alexander Krasichkov, Oleg Markelov, Michael Galagudza, Dmitry Kaplun

**Affiliations:** 1 Department of Micro- and Nanoelectronics, Saint Petersburg Electrotechnical University “LETI”, 197376 Saint Petersburg, Russia; tmzimina@gmail.com (T.Z.); sevostyanov86@bk.ru (E.S.); vvtrushliakova@mail.ru (V.T.); cmid_leti@mail.ru (V.L.); 2 Institute of Highly Pure Biopreparations, 197110 Saint Petersburg, Russia; alexey.kolobov.spb@gmail.com; 3 Radio Engineering Systems Department, Saint Petersburg Electrotechnical University “LETI”, 197376 Saint Petersburg, Russia; askrasichkov@etu.ru; 4 Centre for Digital Telecommunication Technologies, Saint Petersburg Electrotechnical University “LETI”, 5 Professor Popov Street, 197376 Saint Petersburg, Russia; oamarkelov@etu.ru; 5 Almazov National Research Centre, 197341 Saint Petersburg, Russia; galagudza_mm@almazovcentre.ru; 6 Department of Automation and Control Processes, Saint Petersburg Electrotechnical University “LETI”, 197376 Saint Petersburg, Russia

**Keywords:** thick-film technologies, lamination, microfluidics, biosensor, peptide aptamers, dry film photoresist, label-free detection

## Abstract

A study of the peculiarities and a comparative analysis of the technologies used for the fabrication of elements of novel hybrid microfluidic biochips for express biomedical analysis have been carried out. The biochips were designed with an incorporated microfluidic system, which enabled an accumulation of the target compounds in a biological fluid to be achieved, thus increasing the biochip system’s sensitivity and even implementing a label-free design of the detection unit. The multilevel process of manufacturing a microfluidic system of a given topology for label-free fluorometric detection of protein structures is presented. The technological process included the chemical modification of the working surface of glass substrates by silanization using (3-aminopropyl) trimethoxysilane (APTMS), formation of the microchannels, for which SU-8 technologies and a last generation dry film photoresist were studied and compared. The solid-state phosphor layers were deposited using three methods: drop application; airbrushing; and mechanical spraying onto the adhesive surface. The processes of sealing the system, installing input ports, and packaging using micro-assembly technologies are described. The technological process has been optimized and the biochip was implemented and tested. The presented system can be used to design novel high-performance diagnostic tools that implement the function of express detection of protein markers of diseases and create low-power multimodal, highly intelligent portable analytical decision-making systems in medicine.

## 1. Introduction

One of the most dynamically developing areas of bioMEMS/NEMS technologies is that of biosensor devices that are based on spatial molecular recognition and the binding of biological components for the rapid evaluation of physiological parameters, and on this basis, the diagnostics of non-transferred diseases. Modern biosensor systems should provide the necessary sensitivity and selectivity when it comes to analysis, high performance and speed of analysis, small sample volume, portability and low cost [1]. The implementation of this set of characteristics is achieved by a miniature design and by integrating the functional modules into a single heterogeneous system. The structure determines the necessary complexity of the design and technological solutions that ensure both the process of its creation and functioning.

The most important module of the biosensor is the detection system. This system’s working principle and technical implementation largely depend on the chemical structure of the target component of the analysis; in the current case—a globular protein [2,3]. Traditional methods for detecting protein biomarkers, such as solid-phase enzyme-linked immunosorbent assay (ELISA), despite their high sensitivity and selectivity, are quite expensive, time-consuming and multi-stage. The procedures are usually performed in specialized clinical laboratories [4]. Optical and electrochemical detection principles have become the most widely used in miniature devices designed for detecting proteins [5,6], though in most realizations additional specialized labels, such as, immune complexes of fluorescent dyes or other markers are used, complicating the procedure of assay [7]. The development of methods for the direct quantitative registration of proteins recognized and bound to peptide aptamers without the use of complex markers, which complicate and increase the cost of analysis and make it more labor intensive, are important and actual.

Thus, there is a significant interest in the development of simple and reliable detection procedures that are suitable for creating Point-of-care-testing (POCT) devices. A new approach to the label-free registration of protein structures, based on their native fluorescence as described in [8], implies the construction of high-performance intelligent analytical systems for express biomedical analysis, which opens many new possibilities for proteomics and biomedical diagnostics.

An important issue in designing biosensor systems is reducing the sample volume and increasing the performance and throughput of the analysis. Technologies of microfluidics, which allow operative and precise control of laminar flows, open the possibility of increasing the accuracy of sample control with a decrease in the number of reagents [9]. That is why the development of heterogeneous biosensor systems with integrated microfluidic subsystem for efficient manipulation of components of analysis is an important direction of the development of new generation analytical instruments.

One of the most important stages in the development of microanalytical systems is the selection of the optimal material for the device fabrication. In addition to the features of the functioning of the microfluidic system, there are certain necessities to take into account regarding factors such as manufacturability, durability, flexibility, optical transparency, biological and chemical compatibility with intended reagents, compliance with the temperature and pressure conditions required for the reaction, as well as the possibility of surface functionalization [10].

Glass is a chemically inert, thermostable, electrically insulating, mechanically rigid, biologically compatible material, suitable for surface functionalization [10]. These properties allow the use of glass as a substrate for preparation microstructures using chemical reactions requiring extreme conditions: high temperatures; high pressures; and aggressive solvents. Compared to silicon, glass has a significantly higher optical transparency, low price, and wide possibilities for the integration of active components [11]. In addition, in the detection method described in [8], glass works as a filter of ultraviolet radiation, which allows it to re-emit protein fluorescence onto the luminophore layer. The variety of technologies for the formation of microfluidic systems is based on a wide range of materials of an organic and inorganic nature [11,12], which ensures the use of microfluidics as a basic process in hybrid analytical microsystems.

Since microfluidic platforms are quite difficult to clean and are most often used once [13], particularly in POCT applications, an important aspect when choosing their manufacturing technology is the cost and ecological safety. For widespread use, microfluidic chips must be manufactured in a safe, affordable and easily scalable way. At the same time, some authors have noted that microfluidic glass chips can be effectively cleaned and reused [14,15].

Manufacturing technologies for microfluidic devices should be adapted to the characteristics of the material used and the technical requirements for the product, due to the specifics of its application. The most commonly used methods for creating microfluidic structures are photolithographic methods, which are standard technologies in the production of microelectronic and microsystem devices [16]. Another perspective direction is the polymer technologies, including: injection molding; hot embossing; soft lithography, etc. [17]. Notably, there are also the mechanical methods of micromachining that are used to create microfluidic systems. Mechanical processes should provide surface treatment without cracks, while maintaining good dimensional stability of the formed channels and a low level of surface roughness [18]. These methods are suitable for processing silicon and glass, as well as for some polymer materials [19]. Fabrication of laser-ablated and wet-etched devices took approximately 12 and 17 h, respectively, with the bulk of this time dedicated to the bonding of the top cover plate. Nevertheless, the elimination of the photolithography step makes laser ablation a valuable approach to screening multiple revisions of device geometry, without the need to purchase numerous photomasks. Some authors found advantages in quarts chips, since this material is very optically transparent and susceptible to high temperature technologies without cracking [20]. Carbon dioxide (CO_2_)-laser processing of glasses is a versatile mask-less writing technique used to engrave micro-structures and provide a level of flexible control with regard to shape and size. The fabrication of hundreds of microns quartz micro-channels and micro-holes by pulsed CO_2_-laser ablation, with a focus on the huge potential of the technique for use in microfluidics and biomedical applications, is described in [21]. The fabrication was performed in two stages including laser ablation and post-ablation wet etching to remove surface features stemming from laser-texturing that are undesirable for channel sealing [22]. Then, ablated profiles were used to form polymer pillars for cell processing. The use of laser ablation is particularly efficient in prototyping planar microfluidic systems.

The important advantages of methods such as micromilling, abrasive blasting and ultrasonic processing are their low cost, high degree of technological flexibility, and that they can possibly be used in combination with other processes to create complex three-dimensional structures. The main disadvantage of mechanical micromachining processes is their lower accuracy and productivity in comparison with photolithographic methods [10], which ultimately complicates the organization of low-cost widespread production of microfluidic biochips using the detection technique we have developed.

However, in some applications, such as: microbiological analysis; pre-clinical express-testing; and POCT, it is essential that the biosensor is disposable, since there is no possibility to clean and sterilize them.

Of considerable interest is the research and development of the technological foundations of the heterogeneous integration of an artificial biorecognition element that performs the function of spatial recognition of the target structure [8] into the hybrid structure of the biosensor system and ensures a unified integral-group technological cycle of its formation. The atomic-molecular design of a biorecognition element allows the editing of its physical properties in order to improve the functional characteristics of the detection system [23,24,25]. Peptide aptamers (oligopeptides built of 8–40 amino acid residues) can serve as biorecognition elements with a high degree of affinity and a specificity of complex formation with target protein macromolecules. The complexes of peptide aptamers and target proteins have dissociation constants comparable, and are sometimes better than those of antigen-antibody complexes [23]. Therefore, they are extremely promising for the development of biosensors that aim to detect the protein markers of diseases [24,25,26,27].

Thus, this work describes the research and development details and the peculiarities of technologies used for the fabrication of hybrid-integrated planar microfluidic biosensors with peptide recognition elements for the label-free detection system [8].

## 2. Structure and Topology of Functional Components of a Hybrid Biosensor

The main functional components (or subsystems) of hybrid biosensors in development are intended for: biorecognition and selective capture of target proteins; detection of captured proteins; microfluidic transport; casing and inlet/outlet.

**Figure 1:**
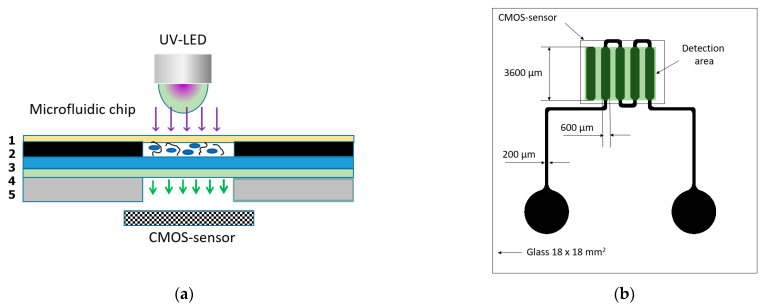
The schematic of biosensor system design and microfluidic system topology. Cross-section view of a sandwich structure of a microfluidic system of biochip with integrated biorecognition elements (**a**): 1—a sealing layer and optical window for inlet UV radiation (polypropylene); 2—a microfluidic system with microchannels and platforms for integrating biorecognition elements (photoresist); 3—a cover glass substrate; 4—a luminophore layer (ZnS: Cu); 5—PETF casing. Topology of a microfluidic system (**b**).

We propose the use of the detection principle presented in Figure 1a, which is based on using a common digital camera with a CMOS matrix, coupled with a microfluidic channel of a biosensor system with biorecognition elements, further coupled with a layer of specific luminophore, as described earlier [8]. The materials within the biosensor, including aptamers, as well as a semiconductor UV radiation source, should not have fluorescence centers [10] as they emit background light under UV excitation.

As most traditional CMOS matrices have a peak response between the green and red regions of the spectrum (λ = 480–560 nm), to efficiently register the native fluorescence of proteins (λ = 300–450 nm), it is necessary to: (1) re-emit it into a longer wavelength region; (2) reduce background fluorescence; (3) focus and accumulate the signal. Both the radiation source and the registering CMOS matrix are united by a low-power electric power supply system through the USB interface. Thus, to achieve these goals, the sandwich-type design of the sensor includes the following layers, from bottom to top (Figure 1a): PETF casing with window (5); luminophore (4); glass substrate with chemically deposited peptide aptamers (3); photoresist profile of the microfluidic system (2); polypropylene film top sealing layer and window for inlet UV radiation (1). In Figure 1b, a basic topology of microfluidic system is presented, including input wells for liquids, a network of microfluidic channels of 200 microns width and 40 microns depth, topologically coded working cells containing immobilized aptamers, and a reference blank working cell for evaluating the background fluorescence level.

Selecting the optimal depth of microchannels compromising the opposite consequences of the capillary cross-section area value is important. We need to take into account the hydraulic resistance, which abruptly reduces the pressure driven volumetric flow velocity, Q, with cross-section area decrease, according to Poiseuille’s law: *Q ≈ r*^4^. On the other hand, the biorecognition layer of immobilized peptides (aptamers) is located on the glass surface of microfluidic channel wall (which serves simultaneously as an optical window and UV filter). The path of the lateral displacement of proteins in the flow should coincide with the channel size in order to ensure the probability of contact and the binding of the proteins by peptide aptamers. In other words, to provide high yield capture of target proteins it is necessary to ensure their diffusion migration towards the surface during the movement of sample along the channel. Thus, it is necessary to select a flow velocity providing a sufficient time for lateral diffusion displacement of proteins across the microfluidic channel of the given size. An estimation of the mean-square displacement (*X*) of protein macromolecules in the liquid phase is possible, using the following expression [28]:

(1) $$X=\sqrt{2Dt},$$

where *D*—the diffusion coefficient of protein particles in the aqueous medium, *t*—time.

Table 1 shows the data calculated by the Formula (1) for the displacement of protein macromolecules of various sizes over a time of 10 s. The diffusion coefficients of proteins are given for an aqueous medium at a temperature of 25 °C [29].

**Figure 2:**
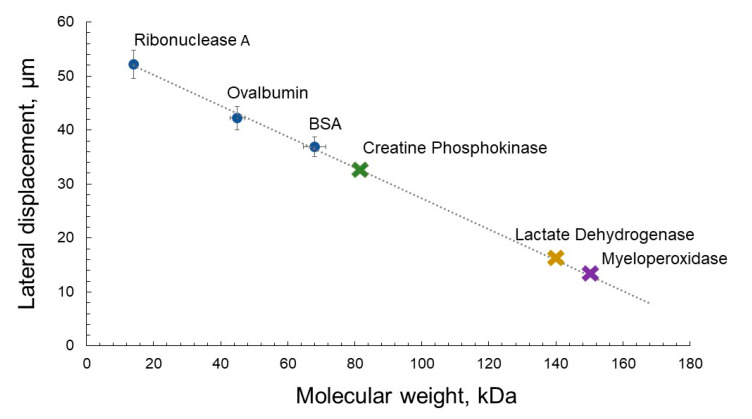
Values of lateral displacement of protein particles depending on the molecular weight of globular proteins.

In Figure 2 relationship of lateral displacement values, evaluated according to Equation (1) versus the molecular weight of globular proteins are presented. A trend shows the displacement for proteins of higher molecular weights, such as myeloperoxidase (*M_W_* = 150 kDa). For this group of larger protein markers, the displacement due to molecular diffusion in 10 s will be about 10 µ. In order to provide a reliable time for all proteins evenly distributed within the channel to reach a surface with immobilized aptamers in 50 µ deep channel, it is necessary that the larger proteins (of up to *M_W_* = 160 kDa) migrate during more than 50 s, according to the evaluation above. During this time, the protein should move within the working area of the microfluidic chip, for example 3600 μm as in the microfluidic system presented here. Thus, the flow velocity in the channel should be <72 μm/s, which is provided by a syringe pump.

**Figure 3:**
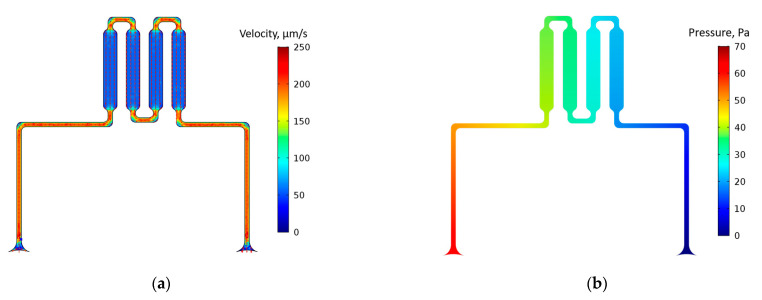
Velocity (**a**) and pressure (**b**) distribution in the microfluidic system.

To visualize the distribution of velocities and pressures in a microfluidic channel, the numerical modeling was carried out using the COMSOL Multiphysics^®^ (COMSOL, Inc., Burlington, MA, USA) code. The simulation was carried out for a laminar flow under stationary conditions with a velocity of 72 µm/s applied at the channel inlet. Figure 3 shows the velocity and pressure distribution in the microfluidic system.

The numerical modelling results presented in Figure 3a show that constant velocity value is formed in the working channel of the microfluidic system, which provides the probability of the capture of proteins by peptide aptamers, covalently bonded to the surface of the channel. The pressure distribution in the microfluidic system is shown in Figure 3b.

The special shape of the interfaces between the straight sections of the channels ensures the uniformity of the flow front along the cross-section and reduces the length of stagnant zones and the effects caused by their presence. The total length of the channels provides, on the one hand, sufficient exposure time, and does not significantly contribute to the overall pressure drop in the system. Thus, the presented topology of the microfluidic system makes it possible to operate not only with small sample volumes and low concentrations of target compounds, but to also be controlled by actuators that do not require the provision of high velocities and pressures in the flow.

## 3. Formation of the Microchannels on a Glass Substrate

The production of a microfluidic system for the biosensor under development is impossible without the integration of a bio-recognizing element capable of selectively binding the target protein into its complementary molecular 3D-site. To do this, it is necessary to immobilize the peptide aptamers on the surface of the detection sites in the microfluidic system, having previously functionalized these sites. This operation can be implemented using the preliminary silanization of glass, which is facilitated by the presence of silanol groups (=Si-OH) on its surface.

Organosilanes are widely used for the creation of coatings in diagnostic applications, synthesis and functionalization of nanoparticles, improvement of material properties and catalysis. They can be used to functionalize various surfaces, such as silicon, titanium oxide or glass [33]. One of the most commonly used organosilanes for the surface functionalization of silicon oxide is (3-aminopropyl) trimethoxysilane (APTMS). The presence of a terminal amino group made it possible to use it as an intermediate layer for binding various organic compounds, including peptide bio-identifying elements, onto the substrate surface.

The stages of silanization of glass substrates are presented in Table 2. The standard cover glasses were used as the glass substrates and simultaneously as optical UV filters. All operations were performed in glass petri dishes on a rocking chair at room temperature. Only one surface of the glasses was turned to the solution all the time. The treatment is reduced to a sequential replacement of solutions and heating of the glasses at the last stage.

The quality control of the silanization process was carried out by atomic force microscopy (AFM) using VEECO Dimension 3100 Atomic Force Microscope (Veeco Instruments, Inc., Plainview, NY, USA). Figure 3 shows AFM images of substrates in contact mode at various stages of processing.

The root-mean-square roughness, *R_q_*, of the untreated glass was 3.08 nm. After washing the glass from organic contaminants, this parameter was 2.01 nm. After the final stage of surface treatment of the substrate by annealing, its root-mean-square roughness was 1.67 nm. The root-mean-square roughness of the surfaces was measured over an area of 5 μm × 5 μm. The values are given by the standard procedure of the AFM instrument.

**Figure 4:**
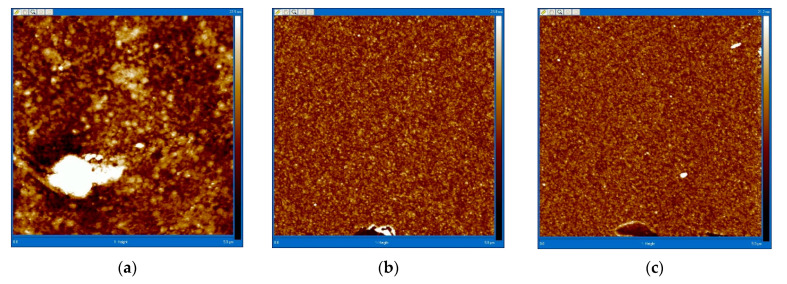

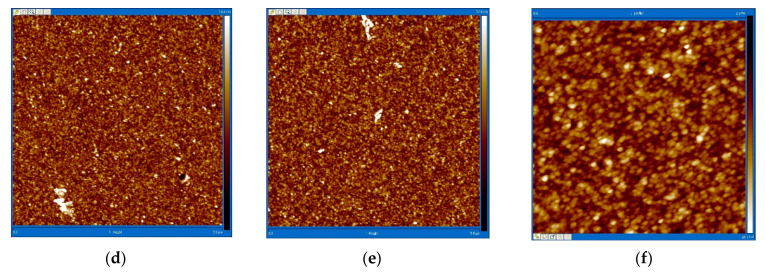
AFM-images of glass substrates at various stages of the silanization process: (**a**)—untreated glass; (**b**)—glass after washing (stage № 1); (**c**)—glass after silanization (stage № 3); (**d**)—glass after washing excess silane layers (stage № 5); (**e**)—glass after annealing (stage № 7); (**f**)—glass with deposited APTMS layer after processing with the dry film photoresist. (**a**–**e**): 5 μm × 5 μm; (**f**): 1.5 μm × 1.5 μm.

AFM images of the surface of glass substrates at different stages of the processing allow the visual changes in the surface of the substrate to be observed, which, together with data on its roughness, allows us to make preliminary conclusions about the successful completion of silanization (Figure 4).

The standard procedure applied in this work to ensure the absence of any residual silanol hydroxyls on the surface of the glass substrate is described below. After cleaning the cover glass plate from contaminants, the surface is covered with free hydroxyl groups. In the next stage—the silanization, the bonding efficiency of silanes, is determined by the excess of APTMS, which was more than 1000 times. After annealing, the surface becomes more uniform, which is demonstrated by the AFM measurements (Figure 4c–e). To increase the efficiency of peptides bonding on amino groups, a 50 times excess of the conjugating reagent—M-maleimidobenzoyl-N-hydroxysuccinimide ester (MBS), was employed. Further, the peptide is deposited on the MBS with an excess of 10 times. Thus, the efficiency of the immobilization process is ensured by a huge excess of reagents. Even if some groups turn out to be freely available for reaction, it will not happen. Only amino groups can remain, and MBS is destroyed in the solution, so nothing else will be bound to it. Thus, as a result of the process, the peptide will either be bound to the surface, or there will be nothing.

We also studied the surface of silanized glass using AFM after the formation of a layer of a microfluidic system from a film photoresist on it (Figure 4f). After the formation of the microfluidic system, the roughness in the selected area was 1.70 nm, which is practically comparable to glass on which the photoresist was not applied. Therefore, the formation of a layer of the microfluidic system after silanization does not violate the structure of the glass surface, on which the immobilization of peptide aptamers are to be immobilized.

A wide range of materials used for the fabrication of microfluidic systems dictates the variety of technologies for their processing. The technologies must be adapted not only to the characteristics of the material used, but also to the technical requirements of the product due to the specifics of its application.

Lithographic methods are widely used for creating profiles of microfluidic structures in solid-state substrates, such as silicon, glass, quarts etc. At the same time, there are examples when a microfluidic system is formed directly from a photoresist layer, for example, from polyimide or SU-8 [34,35]. Photoresist SU-8 is also remarkable due to its resistance to chemicals and solvents. There have been reports [36] that the material proved useful in the fabrication of a microreactor capable of performing radiosynthesis in a quicker, safer and more reliable way compared to traditional vessel-based approaches. There are also known applications of dry film photoresists (DFP) for the fabrication of microfluidic structures [30,37]. These include epoxy-based DFPs as variants of SU-8 (named SUEX). Modern DFPs provide high uniformity of the layer thickness, high aspect ratio of the edges of microchannels, and require fewer technological operations in comparison with liquid photoresists. However, among them, SUEX is costly and chemically too stable for disposable ecologically friendly applications.

**Figure 5:**
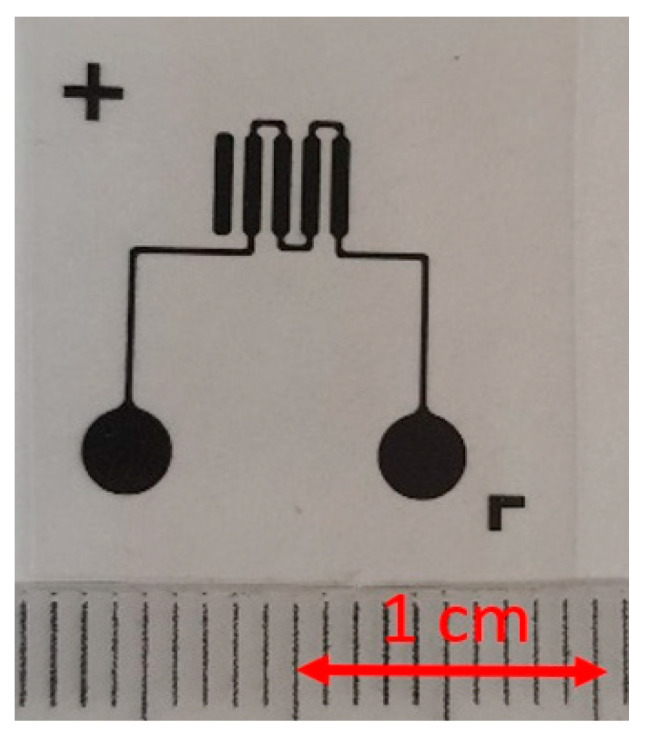
Polymer-based photomask.

In this work, two photoresist materials were studied in order to compare the quality of channels formation of microfluidic systems, adhesion, processing reproducibility, labor intensity and cost. These are negative photoresists SU-8 3050 (Kayaku Advanced Materials, Inc., Westborough, MA, USA) and Ordyl Alpha 350 (ElgaEurope, Nerviano (Milano) Italy). Photoresists were applied onto a clean and/or pre-treated (silanized) glass substrates using a standard procedure recommended by the manufacturer. The topology was transferred using polymer-based photomasks (Figure 5). The print resolution on the polymer substrate of the photomask was 3386 dpi.

The initial option for the microfluidic systems fabrication was SU-8, a high-aspect ratio epoxy-based photoresist developed for microtreatment operations that require a sufficiently thick, chemically and thermally stable coating. This material enables high resolution and aspect ratio microchannels to be formed, particularly on the glass substrates, to which it shows high adhesion level, thereby providing reliable and scalable production processes.

**Figure 6:**
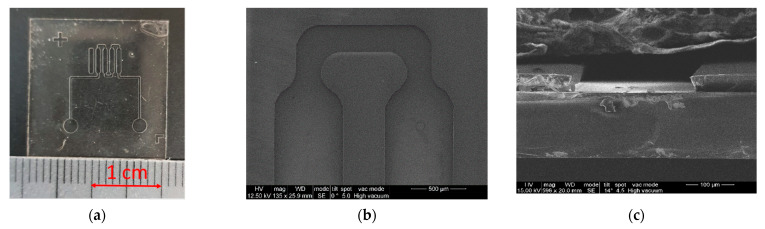
Images of microfluidic systems formed using SU-8 photoresist: (**a**)—general view of the microfluidic system, (**b**)—SEM-image of the detection zone section, (**c**)—SEM-image of the microfluidic channel chip.

The photoresist film was applied by centrifugation at a rotation speed of 900 rpm. After applying the photoresist and removing its excess, it was pre-baked, which made it possible to smooth out the irregularities of the coating and remove unwanted bubbles. As a result, it was possible to obtain a fairly uniform coating with a thickness of about 40 microns. Exposure of the photoresist in ultraviolet radiation was carried out in contact mode using a mercury lamp. After exposure, the photoresist was finally baked for 60 s at a temperature of 95 °C. Then the substrates were placed in a SU-8 Developer (Kayaku Advanced Materials, Inc., Westborough, MA, USA) based on propylene glycol monomethyl ether. After the development, the substrate was washed and dried. SEM images of cross-sections of the microfluidic system profile fabricated using the SU-8 photoresist are shown in Figure 6.

The profiles of microfluidic channels generated with SU-8 photoresist demonstrate the geometry perfectly matching the photomask (Figure 6b) and the 3D structure with high aspect ratio walls (Figure 6c). The channels are perfectly suitable for subsequent processes of assembling a hybrid microfluidic chip and heterogeneous integration of biorecognition elements. Nevertheless, the total production time of test microfluidic structures using SU-8 photoresist turned out to be quite long and labor intensive. In traditional photolithographic methods used to create microfluidic systems based on the SU-8 photoresist technology, the main technological operations were performed for each substrate mainly in an individual mode. In this regard, the creation of 10 samples of chips from SU-8 took more than 3 h. For comparison, it took no more than 1.5 h to create the same number of chips using dry film photoresist technology.

In order to minimize the production time of microfluidic systems and increase the performance of the manufacturing process, the possibility of their formation using a dry film photoresist Ordyl Alpha 350, widely used in the production of printed circuit boards, was investigated. Dry film photoresist Ordyl Alpha 350 have a number of advantages: they provide uniformity and smoothness of the layer; high aspect ratio of walls; require less time to manufacture a microfluidic system; lower cost; and enables fabrication in both a fully equipped cleanroom setting as well as in a minimally equipped laboratory [20].

**Figure 7:**
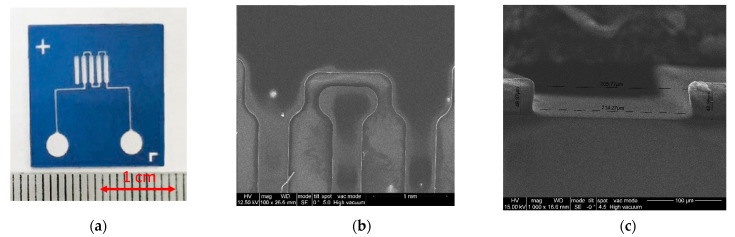
Images of microfluidic system formed using Ordyl Alpha 350 dry film photoresist: (**a**)—general view of the microfluidic system, (**b**)—SEM-image of the detection zone section, (**c**)—SEM-image of the microfluidic channel.

The preparation of substrates for the manufacture of a microfluidic system based on Ordyl Alpha 350 photoresist was carried out by sequential washing with isopropanol and deionized water in an ultrasonic bath for 5 min. After drying, a layer of film photoresist was placed on each of the substrates, completely covering the surface of the substrate. The prepared group of substrates was passed through a MEGA Office PMO 816 laminator at a temperature of 115 °C. After lamination, the samples were exposed to ultraviolet radiation though the mask and processed with sodium carbonate. The images of the microfluidic structure produced using this technological process is shown in Figure 7.

Samples of microfluidic systems formed using the Ordyl Alpha 350 film photoresist technology demonstrated high uniformity of the coating over the entire area of the substrate with an average layer of thickness of about 45 microns, sheer walls and a sufficiently high “contrast” of the shaped elements of the system. The described process made it possible to achieve a sufficiently high quality of the formed elements within the framework of the developed topology and structure of the microfluidic system. The group technology used solves the problem of increasing the productivity of the manufacturing process, which is due to the disposable product requiring production in a sufficiently large number. Film photoresist technology makes it possible to achieve significant volumes of production of microfluidic structures with a simplified version of the technological cycle, which, in turn, ensures its adaptability and integrity within the framework of creating highly intelligent low-power portable express diagnostics systems.

Comparative data on the quality of microfluidic channels fabricated by both methods considered above are presented in Table 3.

Both considered variants of manufacturing technology allow forming channels in layers with approximately the same thickness. The key advantage of the dry film photoresist technology is that it is much more affordable in terms of technology, simplicity and cost, compared to the SU-8 photoresist technology. Based on the analysis of technological processes, the most preferable is the production of microfluidic channels using dry film photoresist Ordyl Alpha 350.

The peptide aptamers used as biorecognition elements are sensitive to the effects of elevated temperatures. Therefore, their immobilization should be carried out after the formation of the microfluidic system, and the sealing of the system should not destroy the structure of the aptamer. The covalent bonding of peptide aptamers was carried out using glutaraldehyde, a homobifunctional crosslinking agent capable of reacting with amino groups. Due to the high specificity of the reaction, the glutaraldehyde method proved efficient in the immobilization of peptides that do not contain reactive groups in the side chain or with only one lysine at the N- or C-terminus. This method is distinguished by its simplicity, speed and efficiency [38]. The essence of the method is reduced to the dissolution of the peptide aptamer and glutaraldehyde in a buffer solution, application of this solution to the silanized glass and rinsing.

## 4. Assembling a Hybrid Biosensor System

After the formation of the microfluidic system channels on a glass substrate and the immobilization of the peptide aptamers, it should be hermetically sealed. This can be done by backing, or by using the glue technologies. In this work we used a UV transparent polypropylene adhesive tape, in which openings for the sample input and output were formed. After merging the adhesive film and the base with a microfluidic system profile, the assembly was consolidated by applying pressure of 1.5 kg/cm^2^ for 2 h. As a result, an optically homogeneous multilayer microchannel composition “glass—photoresist—polypropylene” with integrated peptide aptamers was formed.

**Figure 8:**
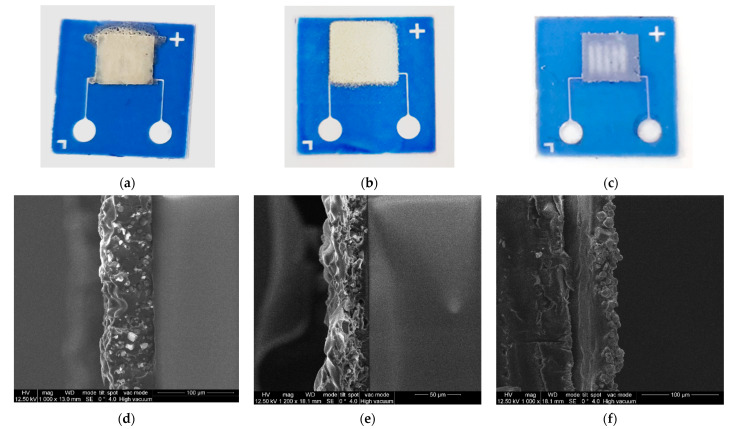
The microfluidic subsystem of the biosensor system with deposited luminophore layer by the following methods: (**a**)—drip application from the liquid phase; (**b**)—spraying from the liquid phase using airbrushing; (**c**)—mechanical spraying on the adhesive surface; SEM images of cross-sections of glass substrates with a luminophore layer; (**d**)—drip application from the liquid phase; (**e**)—spraying from the liquid phase using airbrushing; (**f**)—mechanical spraying on the adhesive surface.

To re-emit the natural fluorescence of protein structures into a longer wavelength region, a layer of luminophore must be applied on the working area of the sealed microfluidic system from the side of the glass substrate/window outer surface. As a result of spectral selection, the ZnS:Cu luminophore of the EL-525 brand with an average grain size of 5–10 microns was selected as a working luminophore [8]. The following methods were studied for applying luminophore (Figure 8a–c): drip application from the liquid phase; spraying from the liquid phase using airbrushing; and mechanical spraying on an adhesive surface. Acrylic varnish was used as a binder for the application methods from the liquid phase. The highest-quality layers in terms of thickness and uniformity were prepared by mechanical spraying onto an adhesive surface. In this case a homogeneous signal was observed at all detection sites.

The thickness of the deposited luminophore layers was estimated using micrometry and scanning electron microscopy of the cross sections of glass substrates. Examples of SEM images of cross-sections of glass substrates with a luminophore layer are shown in Figure 8d–f. It has been established that in the case of drop application, layers with a thickness of about 55–65 µm were obtained, for airbrushing, layers of about 30–35 µm were obtained, and in the case of mechanical spraying onto the adhesive surface, layers were obtained with a thickness of about 25 µm.

**Figure 9:**
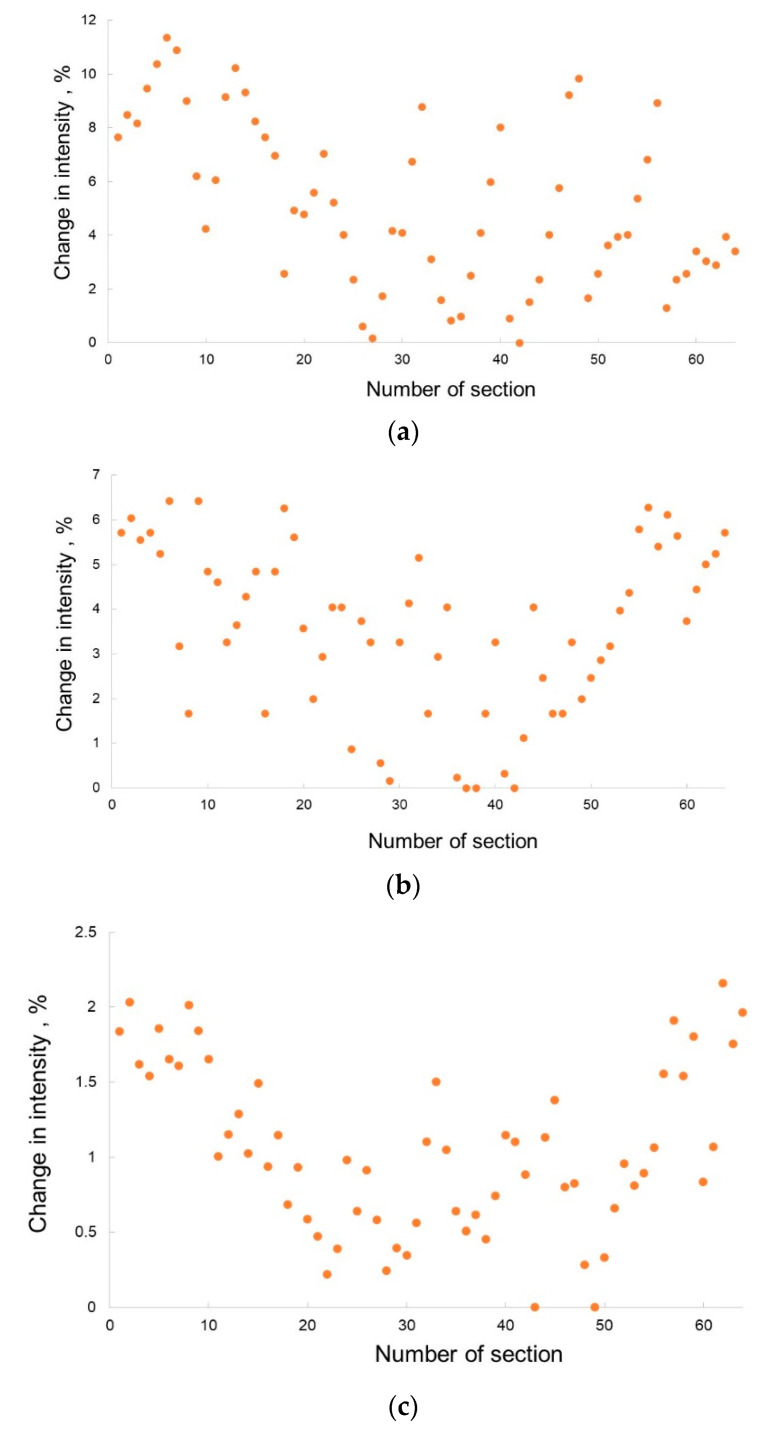
Graphs of the percentage change in intensity relative to the maximum value of the luminescence emission obtained in the selected areas of the sample for different deposition methods: (**a**)—drip application from the liquid phase; (**b**)—spraying from the liquid phase using airbrushing; (**c**)—mechanical spraying on the adhesive surface.

The homogeneity of the luminophore layers was evaluated by measuring the uniformity of the luminescence at different points of the working area of the layer. For this, a UV radiation source with a wavelength close to the wavelength of fluorescence of protein structures was used, namely, an ultraviolet lamp of a DORS 60 viewing detector with a wavelength of 365 nm. The detection area corresponding to the working area of the biochip was divided into 64 sections, and in each of them the luminescence intensity level was measured. Graphs of the percentage change in intensity relative to the maximum value of the luminophore emission obtained in the selected areas of the sample for different deposition methods are shown in Figure 9.

Of the three considered methods, mechanical spraying showed the best results in the homogeneity of the applied luminophore layer: the change in the emission intensity of the luminophore does not exceed 2.5%, while in the case of drop deposition, the intensity of the recorded radiation within one sample can differ by ~12%, and in the case of airbrushing, the limited change in the intensity of the luminophore radiation on the working area of the biochip does not exceed 6.5%.

**Figure 10:**
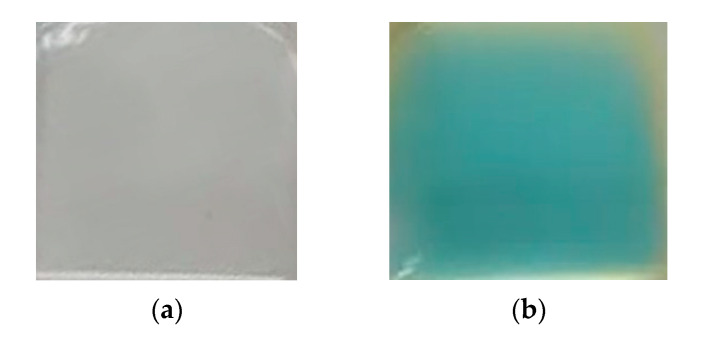
Immunochemical reaction of glass slides treated with antibodies labeled with horseradish peroxidase (HRP), then with HRP chromogenic substrate 3,3′, 5,5′-tetramethylbenzidine (TMB). (**a**)—slide with immobilized Staphylococcus protein A. (**b**)—slide without protein A.

To control the quality of the immobilization technique, an adsorption of protein A on the surface of pure glass cleaned with chromium mixture taken as a negative control (Figure 10a) and glass with APTMS layer (Figure 10b) was used with subsequent binding to antibodies labeled with horseradish peroxidase and then qualitative chemical reaction with 3,3′, 5,5′-tetramethylbenzidine (TMB). The quality of protein immobilization on the glass surface was assessed indirectly by the immunochemical reaction. For that, Staphylococcus protein A was immobilized on the slides surface, then these glasses were treated with antibodies labeled with horseradish peroxidase (HRP) and thoroughly washed. Upon treatment of these slides with HRP chromogenic substrate 3,3′, 5,5′-tetramethylbenzidine (TMB) they developed a blue stain (Figure 10b). This indicates that HRP-labeled antibodies are bound by protein A, and consequently the protein A is bound on the glass. Negative control slides had no protein A on them, but were treated with antibodies the same way. They developed no color reaction after 10 min of exposure to TMB (Figure 10a).

This result also shows that protein A is bound to the glass in the correct orientation, enabling its interaction with antibodies. Thus, we concluded that the method of protein immobilization through APTMS and MBS is suitable for further experiments.

**Figure 11:**
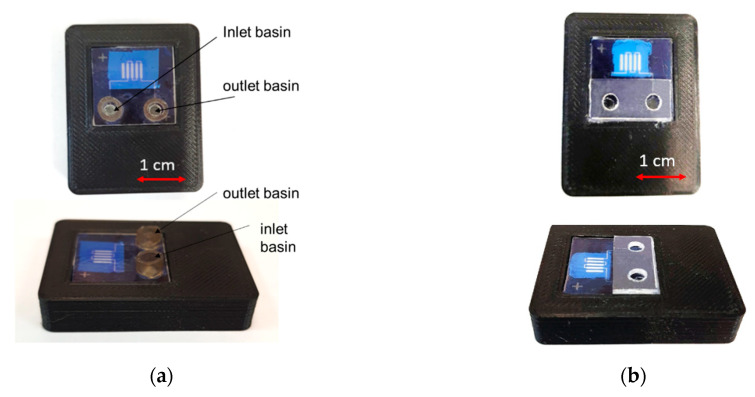
The samples of microfluidic biosensor systems in the assembly: (**a**)—with separate polypropylene input ports; (**b**)—with input ports made of a solid PMMA construction.

After applying the luminophore, the chip was mounted in a case made using three-dimensional printing from polyethylene terephthalate glycol, and cylindrical ports for sample input and output were installed. The connection of the chip to the ports and the case was carried out using a transparent cyanoacrylate glue. The appearance of the assembled microfluidic biosensor systems with various port options is shown in Figure 11.

The use of an adhesive connection made it possible to securely fasten the ports for sample input and output, as well as the biosensor system itself in the case. The resulting designs are hybrid microfluidic biochips created using integrated group technologies that allow them to be scaled for production in large volumes.

**Figure 12:**
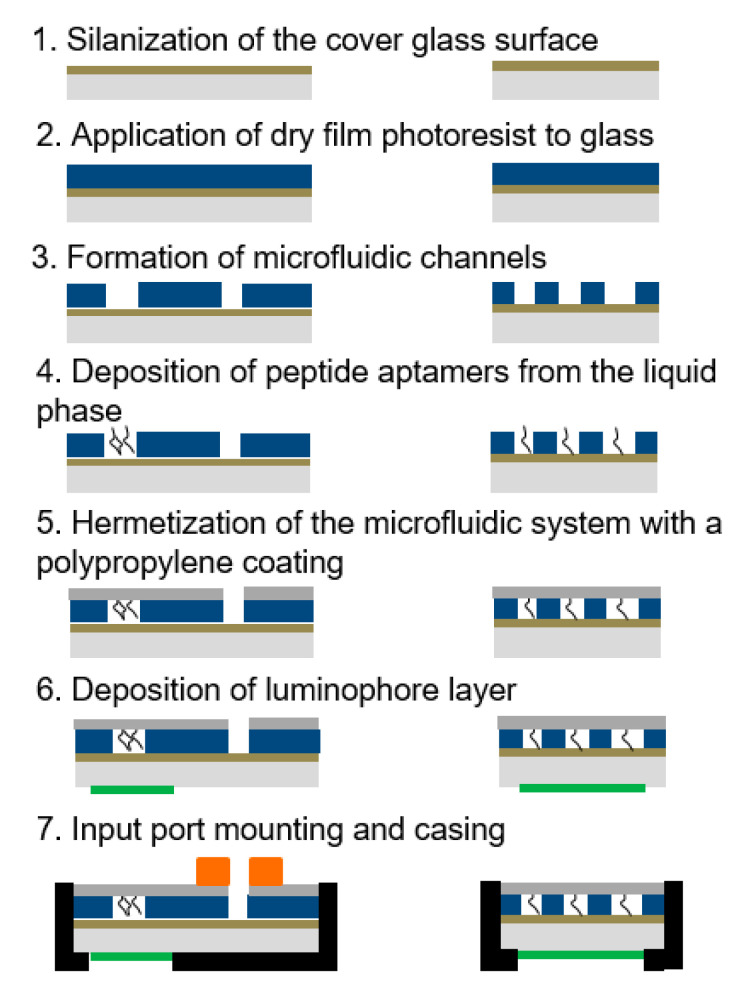
Diagram of the microfluidic biochip manufacturing process, including the stages of heterogeneous integration of a biorecognition element.

The combined technological process of forming hybrid microfluidic biochips, including the stages of heterogeneous integration of bio-recognizing elements into its working area, is schematically presented in Figure 12.

Thus, the production of a hybrid microfluidic biochip under development includes a set of processes that constructively and technologically integrate substances of various physico-chemical nature. Heterogeneous integration of a biorecognition element (proprietary product of the group) is included in the general technological cycle.

## 5. Discussion

The investigation of features and optimization of technologies for the fabrication of hybrid microfluidic biochips aimed towards the label-free detection of protein markers of diseases for implementation of multiparametric (multiple aptamers) express-diagnostics is an extremely actual and important direction of research, which is aimed at the mass-production of economically, materially and energy efficient yet informative disposable microinstruments for express diagnostics of noncommunicable diseases in humans and animals.

The study offers a complex of technological approaches enabling a realization of biosensor systems based on a sandwich geometry and using cost-effective organic and inorganic materials. The optical detection system based on a UV-LED source and a CMOS camera, and the sample control system using a syringe pump, ensures the portability of the developed device and its integrability into modern operating systems. Therefore, such systems, coupled with hybrid-integrated microfluidic biochips operating with small sample volumes, open the prospects for the creation of affordable diagnostic POCT devices to equip the ambulances, clinics and first-aid posts. Due to the novel optical detection principle applied for registration of bound to peptide aptamers target protein-markers, *et specialius,* label-free detection of specifically bound (recognized) target protein-markers, the approach manages to exclude the expensive immune-fluorescent complexes and makes the procedure of analysis easier and faster than traditional ELISA techniques. The total analysis time using the device developed in this work, including sample preparation, takes no more than 8 min. The recognition and immobilization of target protein markers on the peptide aptamers takes no more than 1 min.

However, its implementation exposes requirements to the particular realization of this approach, including a selection of materials with reduced background fluorescence. At the same time, the development of the cost-effective formation of profiles to build the microfluidic system demands the use of specific photoresistive polymeric materials. Thus, the inlet optical window of the system was made of the polypropylene film transparent to UV radiation and not of one exhibiting any fluorescence. The cover-glass substrate exhibits some fluorescence in the blue range, which is filtered. The selection of luminophore is directed in a way so that this optimizes the quantum yield of the system, namely, to absorb light in the region of protein fluorescence, and to emit light in the region of maximal sensitivity of typical web cameras. The deposition of luminophore was a special task, which was aimed at increasing the sensitivity of the system.

**Figure 13:**
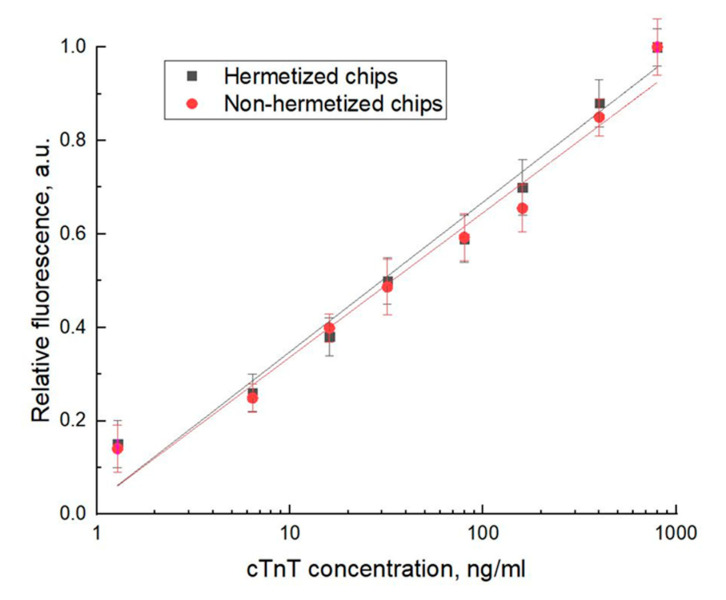
The relationship of relative fluorescence versus concentration of cTnT solutions as measured using microfluidic biochips fabricated according to the described technology for hermetized chips (black line) and non-hermetized chips (red line).

The prepared microfluidic biochips with immobilized peptide aptamers were tested using human Troponin T solutions in the concentration range from 1.28 ng/mL to 800 ng/mL, in a flow-through regime enabling an accumulation of the protein at the working surface of the microfluidic channel with aptamers, thus increasing the concentration sensitivity of the method. Finally, it was possible to reach at the current stage the detection limit of 1.28 ng/mL (Figure 13).

This paper discusses the technological features of the fabrication of hybrid-integrated microfluidic biochips, which were not considered in our earlier work [8], dedicated mostly to the label-free detection technique. We have substantiated an approach to creating disposable biochips based on low cost materials and methods. An improved method of luminophore layer deposition—a mechanical spraying onto an adhesive surface, was described, which provides a considerably more homogeneous structure of the layer. Two photolithographic methods of microchannel profiles formation on the glass surface were considered: using SU-8 epoxy-based negative photoresist and using a dry film photoresist Ordyl Alpha 350. The methods were compared as regards the quality of microchannel profiles, adhesion to glass, number of operations and processing time. We have shown that although rather close to other parameters of the product, the dry film photoresist is considerably more convenient in production, with fewer operations and more simplicity, plus it has the ability to work without the need for clean rooms. Furthermore, the detection limit reported here was decreased [8], by using the concentration of the target molecules on the surface with aptamers by pumping a larger sample volume through the biochip. For example, the detection limit presented in this paper is lower than the one measured on Cobas e411 and i1000SR analyzers, respectively, using the manufacturers calibrators and quality controls. The limit of blank (LoB) of the cTnT assay using those instruments is set to 3 ng/L [39].

In our previous paper [8] we observed the detection limit of 6.5 ng/mL. In the current work due to the improvements in the optical detection system, luminophore deposition technology, accumulation of target molecule possibility and data processing improvement, it was possible to improve the result by about 4 times.

The analytical procedure with the sample biochip included the following stages: sample loading; washing off the access sample; signal capture; and processing. The loading volume of the testing protein solution of human Troponin T in Tris-HCl 0.02 M, pH 7 was 10 μL. The sample was loaded into the inlet basin of the biochip (Figure 11a) and allowed to flow into the capillary channel. Then the inlet basin was connected to the syringe pump and the system was washed with the 100 μL of Tris-HCl 0.02 M buffer solution. The volume necessary for washing the capillary of residual sample was evaluated on the basis of experiments with BSA solutions in untreated glass channels.

The relationship of the fluorescence versus concentration of inlet protein solution was of linear character and obeys the equation: *y* = 0.3202*x* + 0.0279, with *R*^2^ = 0.9785 (Figure 13). The relative values of fluorescence *F_r_* were calculated as: *RFU = (F_S_ − F_B_)/(F_Smax_ −* *F_B_*), where: *F_S_* is the value of the fluorescence signal of the sample, *F_Smax_* is the value of the fluorescence signal of the sample with the maximum measured concentration, *F_B_* is the value of the fluorescence signal of the buffer solution.

Experiments have also been carried out with similar chips, however without a surface coating, to illustrate the contribution of the bulk liquid. The dependence presented in Figure 13 satisfies the linear equation: *y* = 0.3091*x* − 0.0276, with *R^2^* = 0.9774. The signal level was higher up to 10%, which can be attributed to an absence of the sealing coating and to some unevenness of the solution in the open channel. Nevertheless, since we subtract the value of the fluorescence signal of the buffer solution and normalize it relative to the maximum value of the recorded fluorescence, there are no significant differences in the final experimental curves.

The effort enabled a background technological complex to be developed in order to manufacture hybrid microfluidic biochips for label-free detection of protein markers of diseases. Further research will be focused on the design of matrix microfluidic biochips for multiparametric diagnostics on the basis of quantitative detection of diagnostically significant groups of biomarkers. Also, work is in progress now to improve and optimize technological modes and their multiplication for the purpose of mass production.

## 6. Conclusions

The presented technology provides the implementation of a new method of label-free registration of protein markers of diseases for biomedical express analysis [8,10]. The paper discusses the peculiarities of the technology of hybrid microfluidic biochip fabrication, which were not considered in our earlier paper [8]. We have substantiated an approach to creating disposable chips based on cheap construction materials. Ultimately, in this article, in comparison with the previous work [8], an improved view of the device design is presented. For the fabrication of bulk samples, a new technique for deposition of a luminophore layer was developed—mechanical spraying onto an adhesive surface, which provided a considerably more homogeneous layer. This paper also presents new designs for the input and output ports of microfluidic chips. In addition, we managed to achieve an increase in the detection limit by pumping a larger sample volume through the working area of the biochip.

The creation of a hybrid microfluidic biochips for a biosensor system based on the label-free express detection of natural fluorescence of protein structures is presented.

The formation of a microchannels using the Ordyl Alpha 350 film photoresist allows us to achieve a high-performance production of capillary structures of a given topology, thickness, spatial resolution, and a high aspect ratio. The quality is approaching that of SU-8 photoresist, while the production cost and time is considerably lower.

Heterogeneous integration of the bio-identifying element into the microfluidic chip is carried out via preliminary functionalization of the glass substrate by silanol groups using (3-aminopropyl) trimethoxysilane, while the immobilization of the biorecognition element is carried out after the formation of microchannels. This process causes the use of an adhesive polypropylene coating as a sealing layer.

The manufactured biochips are intended for use in biomedical express-diagnostics based on the determination of protein markers of diseases. Biorecognition elements were tested as part of the analysis of the interaction with cardiac Troponin T.

Thus, the presented microfluidic biochip provides heterogeneous integration of biological components with miniature emitting and recording systems, which makes it possible to develop a new effective multiparametric express diagnostic tool.

## Figures and Tables

**Table 1:** Root-mean-square displacement, *X*, over the time of 10 s for protein macromolecules of various molecular weight, *M_W_*, diffusion coefficient, *D*, and hydrodynamic radius, *R_h_*.

Protein	*M_W_*, kDa	*D*, ×10^−11^ m^2^/s	*X*, μm	*R_h_*, nm
Ribonuclease A	14	13.6	52.2	2.65 [30]
Ovalbumin	45	8.9	42.2	3.0 [31]
BSA	68	6.8	36.9	3.5 [32]

**Table 2:** Silanization stages of glass substrate.

№	Operation	Time, Min
1	Washing the glass from organic contaminants and preparing for the application of silanes. The ratio of methanol to hydrochloric acid is 1:1, v/v	30
2	Washing with methanol (2 times for 5 min)	10
3	Silanization with 1% APTMS solution in methanol	60
4	Washing with methanol (2 times for 5 min)	10
5	Washing of excess silane layers to obtain a monolayer using 10% acetic acid solution in methanol	30
6	Washing with methanol (2 times for 5 min)	10
7	Annealing of the sample at temperature of 110 °C so that only covalently bound silanes remain at the surface of substrate	30

**Table 3:** Comparison of parameters of the microfluidic channels formed using SU-8 3050 and Ordyl Alpha 350 photoresists.

Parameter	SU-8 3050	Ordyl Alpha350
High resolution of the generated elements	+	-
The necessity of working in clean rooms	+	+/- *
Good adhesion to the glass substrate	+	+
High uniformity of the layer on the entire surface of the substrate	+/-	+
Toxicity of the developer and photoresist	+	-
The cost of technological equipment and consumables	-	+
The qualification level of technological staff	+	+/-

* Enables fabrication in both a fully equipped cleanroom setting as well as a minimally equipped laboratory depending on the future application.
